# Genome sequence of *Apostasia ramifera* provides insights into the adaptive evolution in orchids

**DOI:** 10.1186/s12864-021-07852-3

**Published:** 2021-07-13

**Authors:** Weixiong Zhang, Guoqiang Zhang, Peng Zeng, Yongqiang Zhang, Hao Hu, Zhongjian Liu, Jing Cai

**Affiliations:** 1 State Key Laboratory of Quality Research in Chinese Medicine, Institute of Chinese Medical Sciences, University of Macau, 999078 Macau, China; 2 Key Laboratory of National Forestry and Grassland Administration for Orchid Conservation and Utilization, 518114 Shenzhen, China; 3 Shenzhen Key Laboratory for Orchid Conservation and Utilization, 518114 Shenzhen, China; 4 National Orchid Conservation Center of China and Orchid Conservation and Research Center of Shenzhen, 518114 Shenzhen, China; 5 Key Laboratory of NFGA for Orchid Conservation and Utilization, Fujian Agriculture and Forestry University, 350002 Fuzhou, China; 6 School of Ecology and Environment, Northwestern Polytechnical University, 710129 Xi’an, China

**Keywords:** Orchidaceae, *Apostasia ramifera*, Comparative genomics, Adaptive evolution

## Abstract

**Background:**

The Orchidaceae family is one of the most diverse among flowering plants and serves as an important research model for plant evolution, especially “evo-devo” study on floral organs. Recently, sequencing of several orchid genomes has greatly improved our understanding of the genetic basis of orchid biology. To date, however, most sequenced genomes are from the Epidendroideae subfamily. To better elucidate orchid evolution, greater attention should be paid to other orchid lineages, especially basal lineages such as Apostasioideae.

**Results:**

Here, we present a genome sequence of *Apostasia ramifera*, a terrestrial orchid species from the Apostasioideae subfamily. The genomes of *A. ramifera* and other orchids were compared to explore the genetic basis underlying orchid species richness. Genome-based population dynamics revealed a continuous decrease in population size over the last 100 000 years in all studied orchids, although the epiphytic orchids generally showed larger effective population size than the terrestrial orchids over most of that period. We also found more genes of the terpene synthase gene family, resistant gene family, and *LOX1*/*LOX5* homologs in the epiphytic orchids.

**Conclusions:**

This study provides new insights into the adaptive evolution of orchids. The *A. ramifera* genome sequence reported here should be a helpful resource for future research on orchid biology.

**Supplementary Information:**

The online version contains supplementary material available at 10.1186/s12864-021-07852-3.

## Background

The Orchidaceae family is one of the largest among flowering plants, with many species exhibiting great ornamental value due to their colorful and distinctive flowers. At present, there are more than 28 000 orchid species assigned to 763 genera [1]. According to their phylogeny, orchids can be divided into five subfamilies, i.e., Apostasioideae, Vanilloideae, Cypripedioideae, Epidendroideae, and Orchidoideae. It has been proposed that whole-genome duplication occurred in the ancestor of all orchid species, which contributed to their survival under significant climatic change [2, 3]. Orchids are a diverse and widespread family of flowering plants. Notably, several orchid species with specialized floral structures, such as labella and gynostemia, appear to have co-evolved with animal pollinators to facilitate reproductive success. In addition to their role in research on evolution and pollination biology, orchids are invaluable to the horticultural industry due to their elegant and distinctive flowers [4].

The genome sequences of several orchid species have been published recently, thereby greatly improving our understanding of orchid biology and evolution. The first reported orchid genome (*Phalaenopsis equestris*) showed evidence of an ancient whole-genome duplication event in the orchid lineage and revealed that expansion of *MADS*-box genes may be related to the diverse morphology of orchid flowers [2]. The subsequent publication of other orchid genome sequences, such as that of *Dendrobium officinale*, *Dendrobium catenatum*, *Phalaenopsis aphrodite*, *Apostasia shenzhenica*, and *Vanilla planifolia*, has provided data for further investigations on the genetic mechanisms underlying orchid species richness [3, 5–8].

The Apostasioideae subfamily consists of terrestrial orchid species [9]. Species within Apostasioideae exhibit various primitive traits, such as radially symmetrical flowers and no labella, supporting the placement of this subfamily as a sister clade to all other orchids [10]. These primitive features are considered ancient characteristics of the orchid lineage [10]. Thus, Apostasioideae species can serve as an important outgroup for evolutionary study of all other orchid subfamilies. Recently, Zhang et al. [3] published the *A. shenzhenica* genome and identified an orchid-specific whole-genome duplication event as well as changes in the *MADS*-box gene family associated with different orchid characteristics. This is the first (and only) genome reported for the Apostasioideae subfamily, with most currently published genomes belonging to the Epidendroideae subfamily. Obtaining genomes for other orchid lineages, especially basal lineages, will greatly facilitate our understanding of orchid evolution. Here, we performed *de novo* assembly and analysis of the *Apostasia ramifera* genome sequence, the second *Apostasia* genome after *A. shenzhenica*. Comparative genomics were carried out with six other published orchid genomes to provide insight into orchid evolution.

## Results

### Genome sequencing and assembly

The genomic DNA of *A. ramifera* was sequenced using the Illumina Hiseq 2000 platform. Sequencing of five libraries with different insert sizes ranging from 250 to 5 000 bp generated more than 57 Gb of clean data, accounting for 156X of the genome sequence (Additional file 1, Table S1). Based on the clean reads, we generated a 365.59-Mb long assembly with a scaffold N50 of 287.45 kb (Table 1 and Additional file 1, Table S2). To assess the quality of the final assembly, clean reads were mapped to the genome sequence, resulting in a mapping ratio of 99.7 %. The completeness of the gene regions in the assembly was examined by BUSCO (Benchmarking Universal Single-Copy Orthologs) assessment [11]. In total, 94.9 % (1 304/1 375) of the universal single-copy orthologs were found in our assembly (Additional file 1, Table S3).

**Table 1:** Statistics related to *A. ramifera* genome assembly

Feature	Summary
Genome Size	365 588 417 bp
Scaffold N50	287 449 bp
Contig N50	30 765 bp
Longest Scaffold	1 388 560 bp
GC Rate	33.38 %
Repeat Content	44.99 %
BUSCO Assessment	94.9 %
Gene Number	22 841

### Genome annotation

Using both *de novo* and library-based repetitive sequence annotation, 164.49 Mb of repetitive elements were uncovered, accounting for 44.99 % of the total assembly (Additional file 1, Table S4). The proportion of repetitive DNA in *A. ramifera* was similar to that in *A. shenzhenica* (43.74 %) but less than that in *P. equestris* (62 %) and *D. catenatum* (78 %). Among the repetitive sequences, transposable elements (TEs) were the most abundant (43.1 %), among which long terminal repeats (LTR) were dominant, accounting for 24.07 % of the total genome (Additional file 1, Table S5 and Fig. S1).

The protein-coding gene models were predicted through a combination of *de novo* and homology-based annotation. In total, 22 841 putative genes were identified in the *A. ramifera* genome, similar to that in *A. shenzhenica* (21 831) but less than that in *V. planifolia* (28 279), *P. equestris* (29 545), and *D. catenatum* (29 257) (Additional file 1, Table S6). Further functional annotation of the predicted genes was carried out by homology searches against various databases, including Gene Ontology (GO), Kyoto Encyclopedia of Genes and Genomes (KEGG), SwissProt, TrEMBL, nr database, and InterPro. Results showed that 19 551 (85.6 %) predicted genes could be annotated (Additional file 1, Table S7). In addition, we identified 40 microRNA, 616 transfer RNA, 1 450 ribosomal RNA, and 108 small nuclear RNA genes in the *A. ramifera* genome (Additional file 1, Table S8).

Synteny comparison based on gene annotations of *A. ramifera* and *A. shenzhenica* identified 927 synteny blocks with an average block size of 12.89 genes (Additional file 1, Table S9). A total of 11 950 gene pairs were covered by these synteny blocks, accounting for 61 and 66 % of the genome sequences of *A. ramifera* and *A. shenzhenica*, respectively (Additional file 1, Table S9). The high co-linearity between their genomes suggested a close relationship between these two species.

### Gene family identification

**Fig. 1:**
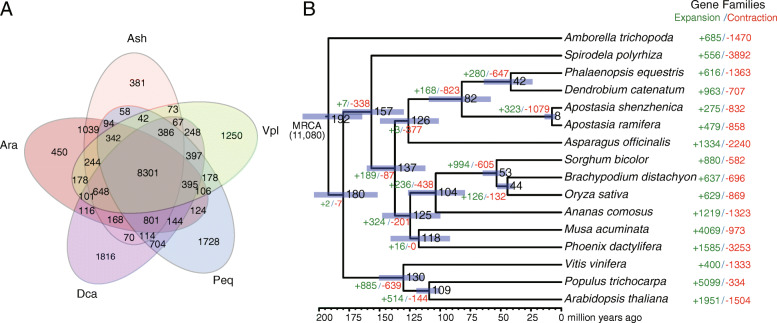
Gene family and phylogenetic relationship analysis. (**A**) Venn diagram showing distribution of shared gene families among five orchid species, i.e., *A. ramifera* (Ara), *A. shenzhenica* (Ash), *P. equestris* (Peq), *D. catenatum* (Dca), and *V. planifolia* (Vpl). (**B**) Phylogenetic tree showing relationship and divergence times for 16 species. Purple bars at internal nodes represent 95 % confidence interval of divergence times. Numbers of expanded and contracted gene families are presented as green and red values, respectively. MRCA, most recent common ancestor

Gene family identification was carried out for the predicted protein-coding genes in *A. ramifera*, together with genes from other species, including *P. equestris*, *P. aphrodite*, *D. officinale*, *D. catenatum*, *A. shenzhenica*, *V. planifolia*, *Asparagus officinalis*, and *Oryza sativa*. A total of 19 422 putative genes in the *A. ramifera* assembly were assigned to 13 251 gene families (Fig. 1 A and Additional file 1, Table S10). The remaining 3 419 genes could not be grouped with other genes and were considered orphans. Among the compared species, 266 gene families were only shared by orchid species. KEGG and GO enrichment analyses of those orchid-specific gene families revealed various significantly enriched pathways and terms, including ‘Stilbenoid, diarylheptanoid and gingerol biosynthesis’ (ko00945), ‘Zeatin biosynthesis’ (ko00908), ‘Flavonoid biosynthesis’ (ko00941), ‘Circadian rhythm - plant’ (ko04712), ‘Regulation of gene expression’ (GO:0010468), and ‘Aromatic compound biosynthetic process’ (GO:0019438) (Additional file 1, Table S11 and S12). Furthermore, a total of 1 145 gene families were specifically expanded in *Apostasia* (see Methods), and were significantly enriched in several pathways, such as ‘Ribosome biogenesis in eukaryotes’ (ko03008), ‘mRNA surveillance pathway’ (ko03015) and ‘Plant-pathogen interaction’ (ko04626) (Additional file 1, Table S13 and S14).

### Phylogenetic analysis

We constructed a phylogenetic tree using MrBayes with gene sequences of 381 single copy genes shared by 16 plant species, including *A. ramifera.* The divergence times among these species were estimated using PAML MCMCTree based on our phylogeny. Results showed that the *Apostasia* species separated from other orchids 82 million years ago (Fig. 1B), consistent with previously published results [3]. The divergence time between *A. ramifera* and *A. shenzhenica* was estimated to be 8 million years ago (Fig. 1B). Gene family expansions and contractions on each phylogenetic branch of the 16 species were estimated using CAFE [12] (Fig. 1B). We further carried out GO/KEGG enrichment analyses on the significantly expanded gene families in *A. ramifera* and found some functionally enriched pathways and terms, including ‘Zeatin biosynthesis’ (ko00908), Glycerophospholipid metabolism (ko00564), ‘Flavin adenine dinucleotide binding’ (GO:0050660), and ‘UDP-N-acetylmuramate dehydrogenase activity’ (GO:0008762) (Additional file 1, Table S15 and S16). In addition, the significantly contracted gene families were enriched in ‘Homologous recombination’ (ko03440), ‘Glycosphingolipid biosynthesis’ (ko00604), ‘Transferase activity, transferring phosphorus-containing groups’ (GO:0016772), and ‘Transferase activity’ (GO:0016740) (Additional file 1, Table S17 and S18).

### History of orchid population size

**Fig. 2:**
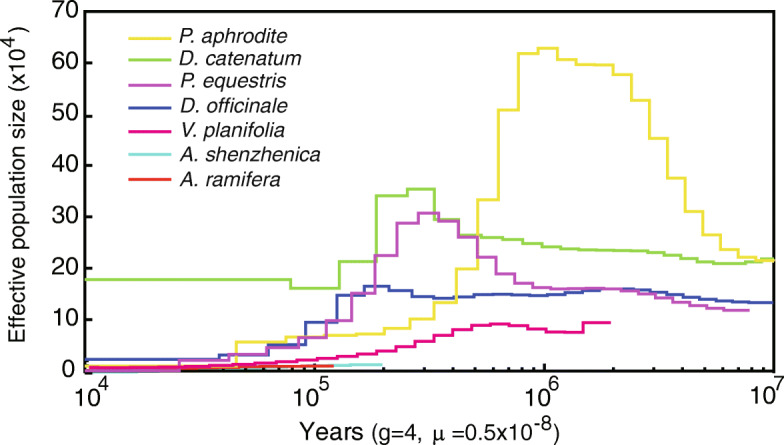
Population size histories of seven orchid species, including *P. aphrodite* (yellow), *D. catenatum* (green), *P. equestris* (purple), *D. officinale* (dark blue), *V. planifolia* (pink), *A. shenzhenica* (light blue) and *A. ramifera* (red), between 10 000 and 10 million years ago. Generation times of orchids were assumed to be four years, and mutation rate per generation was 0.5 × 10^− 8^

Population size history is important for understanding the underlying mechanisms leading to current patterns of species and population diversity [13]. Several investigations on orchid population size have been published [14, 15]. Here, the pairwise sequential Markovian coalescent (PSMC) model, which uses the coalescent approach to estimate population size changes [13], was applied to infer population size history based on the genome sequences of seven orchid species, i.e., *A. ramifera*, *A. shenzhenica*, *P. equestris*, *P. aphrodite*, *D. officinale*, *D. catenatum*, and *V. planifolia*. For the *Apostasia* species, population size changed between 10 000 and 250 000 years ago, with similar population dynamics (Fig. 2). Earlier history could not be recovered because the low-level heterozygosity of the genome sequences of *A. ramifera* and *A. shenzhenica* provided limited information on ancient changes in population size. For the other orchids, population size histories showed similar patterns, especially *D. catenatum*, *D. officinale*, and *P. equestris* (Fig. 2). First, a period of population growth was observed for each of these orchid species. Then, all orchid populations experienced a severe contraction (bottleneck) over the last 100 000 years, from which they have not recovered (Fig. 2). During the reporting period (10 000 to 250 000 years ago), the *Apostasia* species had the smallest population size compared to other orchid species. The population size of *Vanilla* was slightly higher than that of *Apostasia*, but lower than that of all Epidendroideae orchids.

### Gene family evolutionary analysis

#### ***MADS***-box transcription factors

**Fig. 3:**
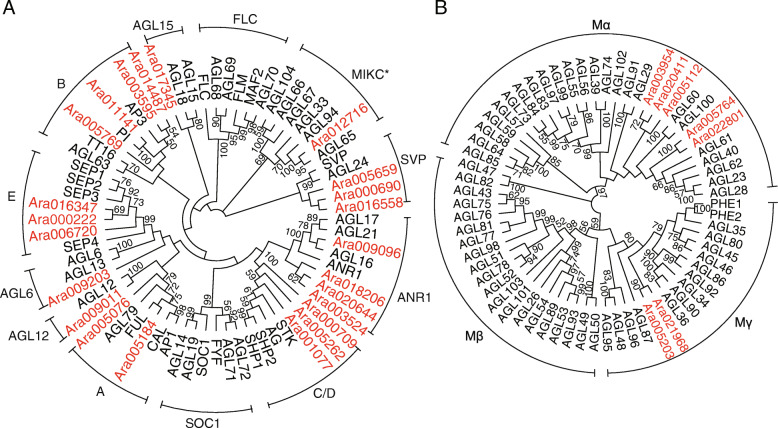
Phylogenetic analysis of *MADS*-box genes in *A. ramifera*. (A) Type II *MADS*-box genes. (B) Type I *MADS*-box genes. Neighbor-joining gene trees were constructed using *MADS*-box genes from *A. ramifera* and *Arabidopsis*. Genes from *A. ramifera* are marked in red. Different *MADS*-box classes are indicated. Numbers above branches are bootstrap support values of at least 50

In plants, *MADS*-box transcription factors are involved in various developmental processes, such as floral development, flowering control, and root growth. All *MADS*-box gene family members are categorized as type I or type II based on their gene tree. Using HMMER software and a *MADS*-box domain profile (PF00319), we identified 30 putative *MADS*-box genes in the *A. ramifera* genome, fewer than that detected in the other sequenced orchids (Additional file 1, Table S19). Phylogenetic analysis of the putative *MADS*-box genes revealed that 23 belonged to the type II *MADS*-box clade (Fig. 3 A), fewer again than that found in other orchids, e.g., *A. shenzhenica* (27 members) [3], *V. planifolia* (30 members, Additional file 1, Fig. S2A), *P. equestris* (29) [2], and *D. catenatum* (35) [5]. Compared to *P. equestris*, there were fewer members in the *A*-class, *B*-class, *E*-class, and *AGL6*-class in *A. ramifera* and *V. planifolia* (Additional file 1, Table S19). In contrast, there were more *SVP*-class, *ANR1*-class, and *AGL12*-class members in *A. ramifera* and *V. planifolia* than in *P. equestris* (Additional file 1, Table S19).

Type I *MADS*-box transcription factors are involved in plant reproduction and endosperm development [16]. Here, we identified seven and six type I *MADS*-box genes in *A. ramifera* and *V. planifolia*, respectively (Fig. 3B and Additional file 1, Fig. S2B and Table S19). Phylogenetic analysis showed that genes in the *Mβ*-class were absent in *A. ramifera* and *V. planifolia*, (Fig. 3B and Additional file 1, Fig. S2B).

#### Terpene synthase (***TPS***) gene family

**Fig. 4:**
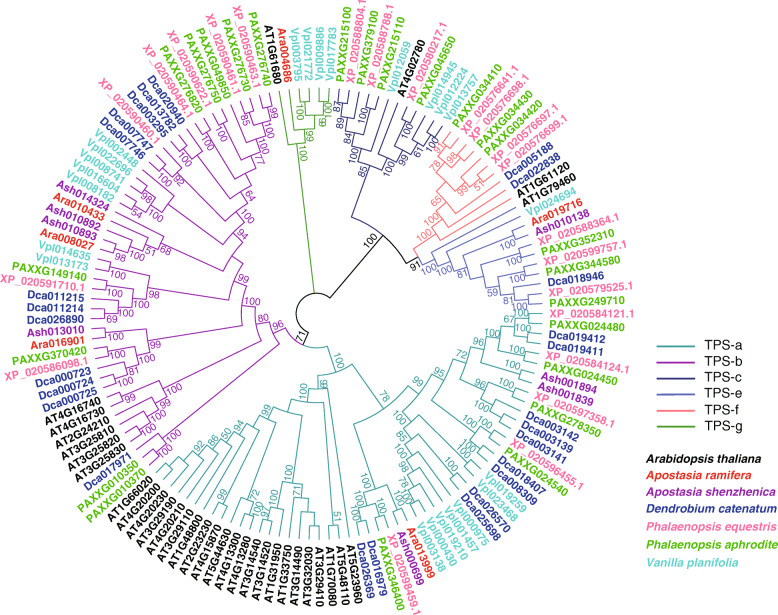
Phylogenetic tree for *TPS* genes predicted in six orchid species and *Arabidopsis*. Numbers above branches are bootstrap support values of at least 50

In plants, *TPS* family members are responsible for the biosynthesis of terpenoids, which are involved in various physiological processes in plants such as primary metabolism and development [17]. The architecture of the *TPS* gene family is proposed to be modulated by natural selection for adaptation to specific ecological niches [18]. We used both terpene_synth and terpene_synth_C domains to search for *TPS* genes in the orchid genomes. A small *TPS* gene family size was observed in the two *Apostasia* species compared with the other orchids studied (Fig. 4). Only eight and six copies of *TPS* genes were found in *A. shenzhenica* and *A. ramifera*, respectively (Fig. 4 and Additional file 1, Table S20). A small *TPS* family size in *Apostasia* may indicate a loss of chemical diversity of terpenoid compounds. To resolve the phylogenetic relationship of *TPS* genes in orchids, a gene tree was constructed using the *TPS* gene sequences derived from orchids and *Arabidopsis*. Phylogenetic analysis showed that four *TPS* subfamilies were found in *Apostasia* (Fig. 4). In *Apostasia*, members of both *TPS*-c and *TPS*-f subfamilies, which encode enzymes responsible for the synthesis of 20-carbon diterpenes, were lost (Fig. 4 and Additional file 1, Table S20). In addition, fewer members of *TPS*-a and *TPS*-b subfamilies were observed in *Apostasia* compared with other orchids (Fig. 4 and Additional file 1, Table S20). Genes from these two subfamilies are reportedly involved in the biosynthesis of 10- and 15-carbon volatile terpenoids [19], which are the components of floral scent.

#### Pathogen resistance genes

**Fig. 5:**
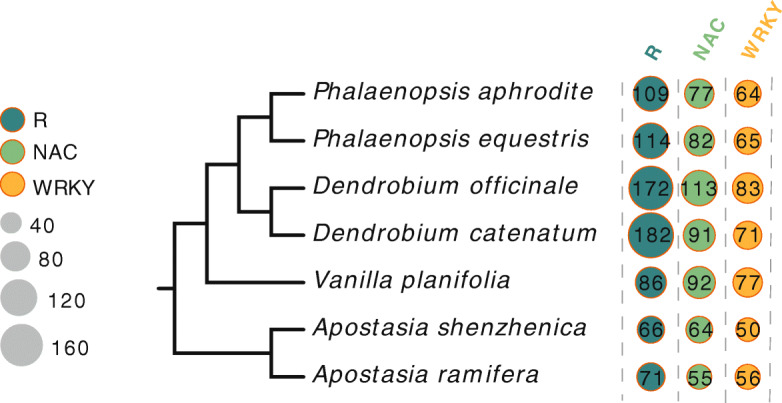
Number of members of *R* genes and *NAC* and *WRKY* gene families in different orchids. These gene families are marked in blue, green, and yellow, respectively. Sizes of circles are directly proportional to number of members in gene family

Pathogen resistance-related genes are closely associated with plant fitness and adaptive evolution [20]. Here, the *NB-ARC* domain profile was used to search for *R* genes in the predicted gene models of *A. ramifera* and other orchids, including *A. shenzhenica*, *V. planifolia*, *P. equestris*, *P. aphrodite*, *D. catenatum*, and *D. officinale*. We identified 71 *R* genes in *A. ramifera* and 66 in *A. shenzhenica*, considerably fewer than that found for *P. equestris* (114), *P. aphrodite* (109), *D. officinale* (172), *D. catenatum* (182), and *V. planifolia* (86) (Fig. 5). Thus, the size of the *R* gene family varied greatly among the different Orchidaceae genera (Fig. 5).

In *Apostasia*, in addition to the small *R* gene family size, we also discovered lower copy numbers in both the *NAC* and *WRKY* gene families (Fig. 5), which are known to play important roles in plant immune response [21, 22]. We identified 55 and 64 *NAC* transcription factor members in *A. ramifera* and *A. shenzhenica*, respectively, markedly fewer than that found in *Dendrobium*, *Phalaenopsis*, and *Vanilla* (77 to 113) (Fig. 5). We also identified 56 and 50 *WRKY* transcription factors in *A. ramifera* and *A. shenzhenica*, respectively, again fewer than that found in other orchids (64 to 83) (Fig. 5).

### ***Apostasia LOX1***/***LOX5*** genes may contribute to lateral root development, an important trait for terrestrial growth

**Fig. 6:**
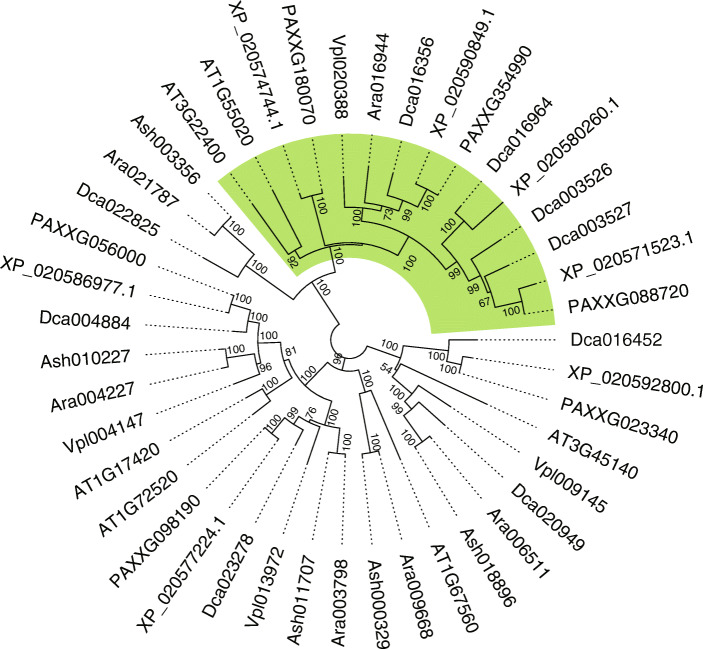
*LOX* gene tree showing *LOX1/LOX5* genes in orchids. Phylogenetic analysis was conducted using *LOX* gene sequences from *A. ramifera*, *A. shenzhenica*, *D. catenatum*, *P. equestris*, *P. aphrodite*, *V. planifolia*, and *Arabidopsis*. Branches leading to orchid *LOX1/LOX5* genes are marked in green. Numbers above branches are bootstrap support values of at least 50

*LOX1* and *LOX5* are involved in the development of lateral roots in *Arabidopsis*, and loss of these two genes causes a significant increase in lateral root emergence [23]. Here, we searched the homologs of *LOX1* and *LOX5* in six published orchid genomes using protein sequences from *Arabidopsis* as the query, and then constructed a gene tree to elucidate the phylogenetic relationship among these genes. We detected multiple copies of *LOX1/LOX5* homologs in the epiphytic orchid genomes (Fig. 6 and Additional file 1, Table S21). However, only one homologous gene was found in *A. ramifera*, and the *LOX1/LOX5* homologs were completely lost in *A. shenzhenica* (Fig. 6 and Additional file 1, Table S21). We also found one copy of the *LOX1/LOX5* genes in the hemi-epiphytic orchid *V. planifolia* (Fig. 6 and Additional file 1, Table S21).

## Discussion

With worldwide distribution, orchids are one of the largest flowering plant families and their extraordinary diversity provides an excellent opportunity to explore plant evolution. Certain evolutionary adaptations in orchids, e.g., pollinium, labella and epiphytism, are proposed to have played key roles in their adaptive evolution and radiation. However, the genetic basis underlying those innovations remains incompletely known. In the current study, we sequenced the genome of *A. ramifera*, a basal Apostasioideae lineage terrestrial orchid, and carried out comparative genomic analyses of seven orchid genomes including that of *A. ramifera*. Several gene families related to adaptations in orchids (e.g., *MADS*-box, pathogen resistance, *TPS*, and *LOX* genes) were compared among different orchid lineages.

### ***MADS***-box transcription factors

Compared with other orchids, we found smaller gene families in the *B*- and *E*-classes of type II *MADS* genes in *Apostasia* and *Vanilla*. Genes in these classes of type II *MADS* are involved in floral development [24]. Furthermore, it has been proposed that small size in these gene families may be related to the maintenance of the ancestral state in *Apostasia* flowers, which exhibit radial symmetry and no specialized labellum [3]. However, small gene families in the *B*- and *E*-classes of the type II *MADS* family were also found in *V. planifolia*, which has bilaterally symmetrical flower petals and a specialized labellum. These results indicate that members in the *B*- and *E*-classes may not contribute to the different flower morphologies found among Apostasioideae and other orchids.

Recent research has suggested that genes from the MIKC* family are involved in pollen development [25, 26]. Here, we found a MIKC* *P*-subclass member in the *A. ramifera* genome. Furthermore, *P*- and *S*-subclasses members of MIKC* were identified in *A. shenzhenica*, while *P*-subclass genes were lost in *P. equestris* [3]. It has been proposed that loss of *P*-subclass genes is associated with the evolution of pollinia [3]. However, both *P*- and *S*-subclass members have been identified in the genome assembly of *P. aphrodite* [7] and *V. planifolia* (Additional file 1, Fig. S2). Thus, loss of MIKC* genes in some orchids might not be relevant to the evolution of pollinia.

A lack of *Mβ* genes has been reported in some orchid genomes, including *A. shenzhenica*, *P. equestris*, and *D. catenatum* [2, 3, 5]. Here, we found that the *Mβ* gene was also absent in *A. ramifera* and *V. planifolia*. Zhang et al. [3] suggested that loss of *Mβ*-class type I *MADS*-box transcription factors is related to the absence of endosperm in the seeds of all orchids. However, *Mβ* genes have been discovered in the genome of *P. aphrodite* and transcriptome of *Orchis italica* [7, 27]. Thus, instead of *Mβ* genes, other genes or mechanisms may contribute to the absence of endosperm in orchid seeds.

### ***TPS*** gene family

In comparison to that in *Apostasia*, more members in the *TPS*-a and *TPS*-b clades of the *TPS* gene family were found in *Vanilla*, *Dendrobium*, and *Phalaenopsis*. Members of these clades are involved in the biosynthesis of volatile terpenoids, which are the components of floral scent [19]. In addition, it has been proposed that expansion of *TPS* subfamilies may promote the emergence of novel compounds [18]. As the flowers of orchids in the Epidendroideae and Vanilloideae subfamilies are highly adapted to animal pollination via many pollination syndromes, including the development of various volatile compounds, this result may provide new insight into the genetic basis of adaptation to insect pollination in epiphytic orchids. Gene duplication and divergence are more effective ways of evolving new enzymatic functions than *de novo* evolution of a new gene [18]. Thus, more members of the *TPS*-a and *TPS*-b subfamilies may facilitate the emergence of novel volatile compounds, which may contribute to their adaptation to diverse animal pollinators via the production of diverse flower scents.

### Lateral root development

For higher land-based plants, roots play a significant role in their successful colonization of the terrestrial environment by providing mechanical support as well as water and nutrient uptake from the soil (or air for epiphytic plants) [28]. Root architecture, i.e., the spatial organization of roots, also has a significant impact on the functional performance of the root system and is important for plant survival [28, 29]. Environmental factors, such as water and nutrient availability, contribute to the shaping of root architecture [30]. Significant root system differences have been reported between *Apostasia* and other orchids [3]. Among them, branch roots have been found in *Apostasia* but not in epiphytic orchids, such as *Phalaenopsis* [3]. In land plants, the formation of lateral roots plays a crucial role in root architecture, uptake, and anchoring. Following their adaptation to soil-free environments, however, various orchids have lost the ability to develop lateral roots, instead forming specialized root structures, such as spongy epidermis, to help preserve nutrients. Zhang et al. [3] reported that variation in the copy number of *ANR1* subfamily *MADS*-box genes results in different lateral root formation between *A. shenzhenica* and epiphytic *P. equestris* and *D. catenatum*. However, as the development of lateral roots is a complicated process that involves intricate regulation and phytohormone interactions [31, 32], the genetic mechanisms controlling the emergence of lateral roots in orchids await further investigation. In this study, we found fewer copies of the *LOX1/LOX5* homologous genes in the *Apostasia* species and hemi-epiphytic *V. planifolia* than that in the epiphytic orchids. Given the function of *LOX1* and *LOX5* in *Arabidopsis* [23], we propose that copy number variation in these genes may contribute to the differences in lateral root development between terrestrial and epiphytic orchids. In addition, according to the phylogenetic relationship of *LOX* genes in orchids, there are six different subclades of *LOX* genes in the common ancestor of orchids. The variation in copy number among the different orchid lineages may be due to the various degrees of gene retention, rather than gene duplication.

## Conclusions

In this study, we performed *de novo* assembly and analysis of the genome of *A. ramifera*, a terrestrial orchid from the Apostasioideae subfamily. We revealed the population size histories of different orchid species and discovered a continuous decrease in population size from the genomes of these species over the last 100 000 years. In addition, the gene family size and subfamily architecture of *TPS* genes varied greatly among species from different orchid subfamilies, which may be associated with the adaptive evolution of orchids. Genes associated with pathogen resistance were significantly reduced in the genomes of *Apostasia* compared with that of other orchids. In *Apostasia*, we also found genes that were likely involved in the regulation of lateral root development, which is an important trait for terrestrial growth. The *A. ramifera* genome sequence reported here should be an important resource for further investigations on orchid biology. Comparative genomics analysis of *A. ramifera* and other orchids should provide new insights into the adaptive evolution of these species.

## Methods

### Sample preparation and sequencing

The *A. ramifera* samples were collected from Jianfeng Mountain, Hainan Province, China. No permission was required to collect these samples. The formal identification of plant material was conducted by Prof. Zhongjian Liu. A voucher specimen of the material was deposited at the National Orchid Conservation Center of China under deposition number Liu.JZ6475. For genome sequencing, we collected fresh leaves from *A. ramifera*. Extraction of genomic DNA was carried out using the modified cetyltrimethylammonium bromide protocol [33]. Five DNA libraries with different insert sizes were constructed using an Illumina library construction kit (NEB DNA Library Rapid Prep Kit for Illumina) and then sequenced using the Illumina HiSeq 2000 platform. After filtering the raw reads according to sequencing quality and adaptor contamination, a total of 57.4 Gb of clean data were retained for assembling the *Apostasia* genome.

### Genome assembly

The estimated genome size of *A. ramifera* was 332.24 Mb according to k-mer frequency distribution. Only one peak was observed in the k-mer distribution, indicating high homozygosity of the *Apostasia* genome. For genome assembly, SOAPdenovo2 [34] was used for contig construction and scaffolding, and GapCloser was used for extending the length of the final contigs. In total, 57.4 Gb of clean reads derived from the DNA libraries with five insert sizes (Additional file 1, Table S1) were used by SOAPdenovo2 assembler and GapCloser for *de novo* genome assembly.

### Repeat annotation

Repeat sequences consist of tandem repeats, such as small and micro-satellite DNA, and interspersed repeats (also known as transposable elements, TEs). In the *A. ramifera* genome, tandem repeat sequences were identified by TRF software [35]. Identification of TEs was conducted by homology searches of the RepBase database [36] and *de novo* prediction. Briefly, RepeatMasker [37] and RepeatProteinMask [38] were applied to identify TEs in the *Apostasia* genome with a RepBase-derived library of known repeat elements. For *de nov*o prediction, we used RepeatModeler and LTR-FINDER [39] to construct a *de novo* repetitive element library for the *A. ramifera* genome. RepeatMasker was then applied to search the genome for TEs with the constructed database. Finally, these results were combined, and the redundant sequences were removed to generate a complete repeat annotation.

### Gene and non-coding RNA prediction

Because previously published work on the *V. planifolia* draft genome [8] did not include gene prediction, we carried out protein-coding gene prediction for the *A. ramifera* and *V. planifolia* genomes. Firstly, we used AUGUSTUS [40] and GlimmerHMM [41] to generate the *de novo* predicted gene sets for our assembly and the *V. planifolia* genome (BioProject: PRJNA507095). Protein sets derived from five plant genomes, including *Arabidopsis thaliana*, *Phalaenopsis equestris*, *Oryza sativa*, *Sorghum bicolor*, and *Zea mays*, were then applied to search against the *Apostasia* and *Vanilla* genomes using TBLASTN with an E-value cutoff of 1e-5 and minimum query coverage of 25 %. GeneWise [42] was used to annotate the gene structures. The RNA-seq datasets (SRR1509356, SRR1509370, and SRR1509674) for *V. planifolia* were downloaded from NCBI SRA, and were *de novo* assembled by Trinity software. *Vanilla* transcripts were applied to annotate the *V. planifolia* genome using the PASA program. The annotation results derived from different methods were then integrated to generate integrated protein-coding gene sets for *A. ramifera* and *V. planifolia* with the MAKER [43] program.

Non-coding RNAs do not translate into protein sequences but exert significant roles in cellular metabolism, and include microRNAs (miRNAs), transfer RNAs (tRNAs), ribosomal RNAs (rRNAs), and small nuclear RNAs (snRNAs). Here, we applied previously described methods to search for non-coding RNAs in the *Apostasia* genome [3]. The miRNA- and snRNA-coding genes were predicted using INFERNAL [44] and the tRNA-coding genes were identified using tRNAscan-SE [45]. Genes encoding rRNAs were annotated by searching the genome with the rRNA sequences of *Arabidopsis*.

### Functional annotation

Functional analysis of the predicted genes in the *Apostasia* genomes was performed by searching their protein-coding regions against sequences derived from publicly available databases, including Gene Ontology (GO) [46, 47], Kyoto Encyclopedia of Genes and Genomes (KEGG) [48], SwissProt [49], TrEMBL [49], non-redundant (nr) protein database, and InterProScan [50].

### Gene family identification

Gene family clustering was conducted using OrthoFinder[51] with complete protein sets from seven species, including *P. equestris*, *P. aphrodite*, *D. officinale*, *D. catenatum*, *A. shenzhenica*, *A. officinalis*, and *O. sativa*, as well as the predicted protein sequences from *A. ramifera*. To limit the disturbance of alternative splicing variants on gene family clustering, the longest transcript of each gene was selected for analysis. Gene families in which the number of genes from *Apostasia* (including *A. ramifera* and *A. shenzhenica*) was 1.5 times higher than that from other orchids were considered expanded in *Apostasia*.

### Phylogenetic analysis

To build a high-confidence phylogenetic tree, we constructed a multi-species protein set containing protein sequences from *A. ramifera* and 15 other species, including 11 monocots (*Spirodela polyrhiza*, *D. catenatum*, *P. equestris*, *A. shenzhenica*, *A. officinalis*, *Ananas comosus*, *Musa acuminata*, *Phoenix dactylifera*, *Brachypodium distachyon*, *S. bicolor*, and *O. sativa*), three eudicots (*Vitis vinifera*, *A. thaliana*, and *Populus trichocarpa*), and the outgroup *Amborella trichopoda*. Protein sequences that contained less than 50 amino acids were removed from the constructed dataset. The pairwise similarities between protein sequences were calculated through all-against-all BLASTP with cutoff criteria: i.e., (i) E-value < 1e-5, (ii) query coverage > 30 %, (iii) alignment identify > 30 %. The results were then entered into OrthoMCL [52] (v2.0.9) to construct orthologous groups. In total, 381 single-copy gene families shared by all 16 species were applied to construct a species tree using MrBayes [53] with the GTR + invgamma model. PAML MCMCTree [54] was used to estimate the species divergence times with the following time calibrations: (i) *O. sativa* and *B. distachyon* divergence time (40–54 million years ago) [55], (ii) *P. trichocarpa* and *A. thaliana* divergence time (100–120 million years ago) [56], (iii) lower boundary of monocot and eudicot divergence time (140 million years ago) [57], and (iv) upper boundary for angiosperm divergence time (200 million years ago) [58]. Gene family expansions or contractions were identified using CAFÉ [12].

### Heterozygosity analysis and estimation of effective population size

Identification of heterozygous loci was performed via a previously described method [59]. Briefly, clean reads were aligned to the genome sequence of *A. ramifera* using the BWA tool [60]. Duplicate reads were then removed by Picard. SAMtools [61] was used for calling heterozygous loci, and bcftools was used for generating consensus sequences. The effective population sizes of the orchid species were estimated using the PSMC program [13]. The parameters for PSMC analysis were set to default except for -g 4 and -u 0.5 × 10^− 8^.

### Identification of ***MADS***-box, ***TPS***, ***NAC***, ***WRKY***, ***R***, and ***LOX*** genes

The hidden Markov model profiles [62] were applied to search for *MADS*-box (Pfam Accession: PF00319), *TPS* (Pfam Accession: PF01397 and PF03936), *NAC* (Pfam Accession: PF02365), *WRKY* (Pfam Accession: PF03106), and *R* (Pfam Accession: PF00931) genes using HMMER [63] (v3.2.1). *MADS*-box genes in *A. thaliana* reported in [3] were used to reconstruct gene trees with the *MADS*-box genes identified in *A. ramifera* and *V. planifolia*. EvolView [64] was used to visualize the number of members in the *NAC*, *WRKY* and *R* gene families for the selected species. For the *TPS* genes, the protein sequences that possessed both Pfam domains and contained more than 500 amino acids were considered as functional genes and used for further analysis. To identify *LOX* genes, protein sequences of the *LOX* gene family in *A. thaliana* (Gene ID: AT1G55020, AT1G72520, AT1G67560, AT1G17420, AT3G22400, and AT3G45140) were used to search for homologous genes in orchids. The identified protein sequences of each gene family were aligned using MUSCLE [65] (v3.8.31) with default settings. MEGA7 [66] was then used to construct an unrooted neighbor-joining tree for each gene family with 500 bootstrap replicates.

## Supplementary Information

**Additional file 1.**

## Data Availability

Raw data and the genome assembly from this study were deposited in NCBI under the BioProject ID: PRJNA635894. The datasets supporting the conclusions of this article are included within the article and its additional files.

## Abbreviations

TEs: Transposable elements.
GO: Gene Ontology.
KEGG: Kyoto Encyclopedia of Genes and Genomes.
PSMC: Pairwise sequential Markov coalescent.
TPS: Terpene synthase.
