# Supplementary material for: Genome sequence of Apostasia ramifera provides insights into the adaptive evolution in orchids
Source: BMC Genomics. 2021 Jul 13;22:536. doi: 10.1186/s12864-021-07852-3 (PMC8278605; doi:10.1186/s12864-021-07852-3)
Supplement: Supplementary file 1 — Additional file 1. [file 12864_2021_7852_MOESM1_ESM.doc]

**Supplementary information**

Table S1 Sequencing libraries of *A. ramifera* genome

| Insert Size (bp) | Read length (bp) | Number of reads | Total bases (bp) | |
| --- | --- | --- | --- | --- |
| 250 | 150 | 69 691 700 | 20 907 510 000 | |
| 500 | 90 | 69 468 117 | 12 504 261 060 |  |
| 800 | 90 | 60 317 166 | 10 857 089 880 |  |
| 2 000 | 90 | 52 024 330 | 9 364 379 400 |  |
| 5 000 | 90 | 20 788 650 | 3 741 957 000 |  |
| Total |  |  | 57 375 197 340 |  |

Table S2 Statistics related to *A. ramifera* assembly

|  | Scaffold length (bp) | Number | Contig length (bp) | Number | |
| --- | --- | --- | --- | --- | --- |
| Max length | 1 388 560 |  | 231 551 |  | |
| N10 | 686 223 | 43 | 81 298 | 339 |  |
| N20 | 548 784 | 103 | 59 948 | 859 |  |
| N30 | 435 934 | 179 | 47 582 | 1 533 |  |
| N40 | 357 602 | 271 | 38 250 | 2 371 |  |
| N50 | 287 449 | 386 | 30 765 | 3 415 |  |
| N60 | 224 942 | 528 | 23 910 | 4 730 |  |
| N70 | 166 362 | 716 | 17 625 | 6 466 |  |
| N80 | 110 565 | 985 | 11 253 | 8 975 |  |
| N90 | 39 071 | 1 494 | 3 527 | 14 181 |  |
| Total length | 365 588 417 |  | 357 649 306 |  |  |

Table S3 BUSCO assessment results

| **Type** | **Number** | **Percentage (%)** |
| --- | --- | --- |
| Complete BUSCOs (C) | 1249 | 90.9 |
| Complete and single-copy BUSCOs (S) | 1237 | 90.0 |
| Complete and duplicated BUSCOs (D) | 12 | 0.9 |
| Fragmented BUSCOs (F) | 55 | 4.0 |
| Missing BUSCOs (M) | 71 | 5.1 |
| Total BUSCO groups searched | 1375 | 100 |

Table S4 Statistics related to repeat sequences in *A. ramifera* genome

| Method | Repeat size (bp) | % of Genome | |
| --- | --- | --- | --- |
| TRF | 19 114 234 | 5.23 | |
| RepeatMasker | 30 885 198 | 8.45 |  |
| RepeatProteinMask | 35 027 408 | 9.58 |  |
| De novo | 143 769 299 | 39.33 |  |
| Total | 164 491 340 | 44.99 |  |

Table S5 Summary of transposable elements in *A. ramifera* genome

|  | Length (bp) | % in Genome | |
| --- | --- | --- | --- |
| DNA | 25 295 929 | 6.92 | |
| LINE | 39 302 569 | 10.75 |  |
| SINE | 17 960 | 0.00 |  |
| LTR | 87 995 459 | 24.07 |  |
| Other | 4 780 | 0.00 |  |
| Unknown | 17 049 310 | 4.66 |  |
| Total | 157 603 083 | 43.11 |  |

Table S6 Comparison of gene features among four orchid species

| Feature | *A. ramifera* | *A. shenzhenica* | *D. catenatum* | *P. equestris* | *V. planifolia* |
| --- | --- | --- | --- | --- | --- |
| Gene number | 22 841 | 21 831 | 29 257 | 29 545 | 28 279 |
| Average mRNA length | 6 497.07 | 7 104.63 | 8 616.36 | 8 964.19 | 7 812.59 |
| Average CDS length | 1 031.14 | 1 100.07 | 1 011.17 | 841.79 | 1 150.45 |
| Exon number per gene | 4.23 | 4.51 | 3.85 | 3.57 | 5.10 |
| Average exon length | 243.53 | 244.06 | 262.45 | 236.00 | 225.53 |
| Average intron length | 1 689.67 | 1 436.57 | 2 385.60 | 2 934.25 | 1 543.23 |
| Exon number | 96 712 | 98 402 | 112 722 | 105 386 | 144 253 |
| Intron number | 73 871 | 76 571 | 83 465 | 75 841 | 122 081 |
| Intron length | 124 817 423 | 109 999 765 | 199 114 351 | 222 536 403 | 188 398 762 |

Table S7 Functional annotation results of predicted genes

|  |  | Number | Percentage (%) |
| --- | --- | --- | --- |
| Gene number |  | 22 841 |  |
| annotated | InterPro | 15 005 | 65.69 |
|  | GO | 10 693 | 46.81 |
|  | KEGG | 10 983 | 48.08 |
|  | SwissProt | 12 593 | 55.13 |
|  | TrEMBL | 19 477 | 85.27 |
|  | nr | 19 551 | 85.60 |
| Unannotated |  | 3 290 | 14.40 |

Table S8 Annotation results related to non-coding RNAs in *A. ramifera* genome

| Type |  | Number | Average length (bp) | Total length (bp) | % of Genome |
| --- | --- | --- | --- | --- | --- |
| miRNA |  | 40 | 123.50 | 4 940 | 0.001351 |
| tRNA |  | 616 | 74.27 | 45 753 | 0.012515 |
| rRNA | rRNA | 1 450 | 130.27 | 188 889 | 0.051667 |
|  | 18S | 58 | 719.64 | 41 739 | 0.011417 |
|  | 28S | 147 | 141.22 | 20 760 | 0.005679 |
|  | 5.8S | 25 | 136.44 | 3 411 | 0.000933 |
|  | 5S | 1 220 | 100.80 | 122 979 | 0.033639 |
|  | 8S | 0 | 0 | 0 | 0 |
| snRNA | snRNA | 108 | 106.57 | 11 510 | 0.003148 |
|  | CD-box | 55 | 105.16 | 5 784 | 0.001582 |
|  | HACA-box | 0 | 0 | 0 | 0 |
|  | splicing | 53 | 108.04 | 5 726 | 0.001566 |
|  | scaRNA | 0 | 0 | 0 | 0 |

Table S9 Co-linear blocks between *A. ramifera* and *A. shenzhenica*

| Species A | Species B | Number of co-linear blocks | Gene pairs in syntenic blocks | Total length of blocks in species A | Total length of blocks in species B |
| --- | --- | --- | --- | --- | --- |
| *A. ramifera* | *A. shenzhenica* | 927 | 11 950 | 61% | 66% |

Table S10 Gene family analysis of seven orchid species

| Species | Gene number | Number of genes in gene family | Number of unassigned genes | Number of gene families |
| --- | --- | --- | --- | --- |
| *A. ramifera* | 22 841 | 19 422 | 3 419 | 13 251 |
| *A. shenzhenica* | 21 831 | 19 957 | 1 874 | 12 828 |
| *D. officinale* | 35 567 | 31 675 | 3 892 | 15 577 |
| *D. catenatum* | 29 257 | 26 690 | 2 567 | 14 476 |
| *P. aphrodite* | 28 910 | 27 087 | 1 823 | 13 542 |
| *P. equestris* | 29 545 | 26 062 | 3 483 | 13 914 |
| *V. planifolia* | 28 279 | 23 869 | 4 410 | 12 956 |

Table S11 Enriched GO terms in gene families exclusively shared by orchids

| GO ID | GO term | GO class | *P* value |
| --- | --- | --- | --- |
| GO:0006355 | regulation of transcription, DNA-templated | BP | 3.96E-4 |
| GO:0051252 | regulation of RNA metabolic process | BP | 3.96E-4 |
| GO:1903506 | regulation of nucleic acid-templated transcription | BP | 3.96E-4 |
| GO:2001141 | regulation of RNA biosynthetic process | BP | 3.96E-4 |
| GO:0019219 | regulation of nucleobase-containing compound metabolic process | BP | 4.68E-4 |
| GO:0010556 | regulation of macromolecule biosynthetic process | BP | 4.88E-4 |
| GO:2000112 | regulation of cellular macromolecule biosynthetic process | BP | 4.88E-4 |
| GO:0009889 | regulation of biosynthetic process | BP | 5.09E-4 |
| GO:0031326 | regulation of cellular biosynthetic process | BP | 5.09E-4 |
| GO:0010468 | regulation of gene expression | BP | 5.19E-4 |
| GO:0051171 | regulation of nitrogen compound metabolic process | BP | 5.53E-4 |
| GO:0080090 | regulation of primary metabolic process | BP | 6.50E-4 |
| GO:0055114 | oxidation-reduction process | BP | 6.92E-4 |
| GO:0060255 | regulation of macromolecule metabolic process | BP | 7.18E-4 |
| GO:0031323 | regulation of cellular metabolic process | BP | 7.32E-4 |
| GO:0006351 | transcription, DNA-templated | BP | 7.71E-4 |
| GO:0097659 | nucleic acid-templated transcription | BP | 7.71E-4 |
| GO:0032774 | RNA biosynthetic process | BP | 7.85E-4 |
| GO:0019222 | regulation of metabolic process | BP | 1.69E-3 |
| GO:0034654 | nucleobase-containing compound biosynthetic process | BP | 2.56E-3 |
| GO:0050794 | regulation of cellular process | BP | 3.53E-3 |
| GO:0065007 | biological regulation | BP | 5.60E-3 |
| GO:0016567 | protein ubiquitination | BP | 6.08E-3 |
| GO:0032446 | protein modification by small protein conjugation | BP | 6.08E-3 |
| GO:0070647 | protein modification by small protein conjugation or removal | BP | 6.08E-3 |
| GO:0019438 | aromatic compound biosynthetic process | BP | 6.25E-3 |
| GO:0050789 | regulation of biological process | BP | 6.86E-3 |
| GO:0018130 | heterocycle biosynthetic process | BP | 7.17E-3 |
| GO:0016070 | RNA metabolic process | BP | 9.40E-3 |
| GO:1901362 | organic cyclic compound biosynthetic process | BP | 9.47E-3 |
| GO:0009240 | isopentenyl diphosphate biosynthetic process | BP | 1.44E-2 |
| GO:0019288 | isopentenyl diphosphate biosynthetic process, methylerythritol 4-phosphate pathway | BP | 1.44E-2 |
| GO:0046490 | isopentenyl diphosphate metabolic process | BP | 1.44E-2 |
| GO:0009607 | response to biotic stimulus | BP | 1.68E-2 |
| GO:0006952 | defense response | BP | 2.09E-2 |
| GO:0000902 | cell morphogenesis | BP | 2.87E-2 |
| GO:0008360 | regulation of cell shape | BP | 2.87E-2 |
| GO:0022604 | regulation of cell morphogenesis | BP | 2.87E-2 |
| GO:0032989 | cellular component morphogenesis | BP | 2.87E-2 |
| GO:0010467 | gene expression | BP | 3.19E-2 |
| GO:0044271 | cellular nitrogen compound biosynthetic process | BP | 4.27E-2 |
| GO:0048869 | cellular developmental process | BP | 4.27E-2 |
| GO:0008171 | O-methyltransferase activity | MF | 4.97E-14 |
| GO:0008762 | UDP-N-acetylmuramate dehydrogenase activity | MF | 2.66E-10 |
| GO:0005488 | binding | MF | 1.28E-07 |
| GO:0050660 | flavin adenine dinucleotide binding | MF | 6.40E-07 |
| GO:0008168 | methyltransferase activity | MF | 1.89E-05 |
| GO:0010333 | terpene synthase activity | MF | 3.80E-05 |
| GO:0016741 | transferase activity, transferring one-carbon groups | MF | 4.29E-05 |
| GO:0016616 | oxidoreductase activity, acting on the CH-OH group of donors, NAD or NADP as acceptor | MF | 6.35E-05 |
| GO:0046983 | protein dimerization activity | MF | 7.08E-05 |
| GO:0016838 | carbon-oxygen lyase activity, acting on phosphates MF | MF | 7.49E-05 |
| GO:0046872 | metal ion binding | MF | 1.33E-4 |
| GO:0043169 | cation binding | MF | 1.59E-4 |
| GO:0016614 | oxidoreductase activity, acting on CH-OH group of donors | MF | 1.96E-4 |
| GO:0005506 | iron ion binding | MF | 4.35E-4 |
| GO:0016705 | oxidoreductase activity, acting on paired donors, with incorporation or reduction of molecular oxygen | MF | 1.33E-3 |
| GO:0043167 | ion binding | MF | 1.77E-3 |
| GO:0050662 | coenzyme binding | MF | 1.79E-3 |
| GO:0001071 | nucleic acid binding transcription factor activity | MF | 1.97E-3 |
| GO:0003700 | transcription factor activity, sequence-specific DNA binding | MF | 1.97E-3 |
| GO:0020037 | heme binding | MF | 2.10E-3 |
| GO:0046906 | tetrapyrrole binding | MF | 2.17E-3 |
| GO:0009055 | electron carrier activity | MF | 2.78E-3 |
| GO:0016835 | carbon-oxygen lyase activity | MF | 5.75E-3 |
| GO:0005515 | protein binding | MF | 8.74E-3 |
| GO:0048037 | cofactor binding | MF | 1.07E-2 |
| GO:0003720 | telomerase activity | MF | 1.73E-2 |
| GO:0003721 | telomerase RNA reverse transcriptase activity | MF | 1.73E-2 |
| GO:0008766 | UDP-N-acetylmuramoylalanyl-D-glutamyl-2,6-diaminopimelate-D-alanyl-D-alanine ligase activity | MF | 1.73E-2 |
| GO:0016708 | oxidoreductase activity, acting on paired donors, with incorporation or reduction of molecular oxygen, NAD(P)H as one donor, and incorporation of two atoms of oxygen into one donor | MF | 1.73E-2 |
| GO:0004842 | ubiquitin-protein transferase activity | MF | 2.44E-2 |
| GO:0019787 | ubiquitin-like protein transferase activity | MF | 2.69E-2 |
| GO:0000287 | magnesium ion binding | MF | 3.56E-2 |
| GO:0046914 | transition metal ion binding | MF | 4.37E-2 |
| GO:0016491 | oxidoreductase activity | MF | 4.38E-2 |
| GO:0000151 | ubiquitin ligase complex | CC | 1.26E-3 |
| GO:1990234 | transferase complex | CC | 2.52E-3 |
| GO:0000145 | exocyst | CC | 8.88E-3 |
| GO:0005938 | cell cortex | CC | 8.88E-3 |
| GO:0044448 | cell cortex part | CC | 8.88E-3 |
| GO:0099568 | cytoplasmic region | CC | 8.88E-3 |
| GO:1902494 | catalytic complex | CC | 1.37E-2 |
| GO:0044391 | ribosomal subunit | CC | 1.95E-2 |
| GO:0071944 | cell periphery | CC | 4.62E-2 |
| GO:0005759 | mitochondrial matrix | CC | 4.77E-2 |
| GO:0005787 | signal peptidase complex | CC | 4.77E-2 |

Table S12 Enriched KEGG pathways in gene families exclusively shared by orchids

| Map ID | Map Title | *P* value |
| --- | --- | --- |
| map00945 | Stilbenoid, diarylheptanoid and gingerol biosynthesis | 1.29E-15 |
| map00908 | Zeatin biosynthesis | 1.97E-07 |
| map00941 | Flavonoid biosynthesis | 5.72E-05 |
| map00943 | Isoflavonoid biosynthesis | 1.97E-4 |
| map00902 | Monoterpenoid biosynthesis | 8.99E-4 |
| map04712 | Circadian rhythm - plant | 1.23E-3 |

*Table S13 Enriched GO terms in expanded gene families in* Apostasia

| GO ID | GO term | GO class | *P* value |
| --- | --- | --- | --- |
| GO:0006259 | DNA metabolic process | BP | 2.05E-96 |
| GO:0006278 | RNA-dependent DNA replication | BP | 2.61E-91 |
| GO:0006260 | DNA replication | BP | 2.03E-77 |
| GO:0090304 | nucleic acid metabolic process | BP | 1.55E-52 |
| GO:0015074 | DNA integration | BP | 6.36E-47 |
| GO:0006139 | nucleobase-containing compound metabolic process | BP | 1.12E-44 |
| GO:0046483 | heterocycle metabolic process | BP | 4.65E-42 |
| GO:0006725 | cellular aromatic compound metabolic process | BP | 1.58E-41 |
| GO:1901360 | organic cyclic compound metabolic process | BP | 5.28E-40 |
| GO:0034641 | cellular nitrogen compound metabolic process | BP | 3.66E-33 |
| GO:0006807 | nitrogen compound metabolic process | BP | 2.12E-30 |
| GO:0043170 | macromolecule metabolic process | BP | 6.45E-28 |
| GO:0044260 | cellular macromolecule metabolic process | BP | 3.93E-27 |
| GO:0034645 | cellular macromolecule biosynthetic process | BP | 5.60E-25 |
| GO:0009059 | macromolecule biosynthetic process | BP | 7.44E-25 |
| GO:0044249 | cellular biosynthetic process | BP | 1.36E-15 |
| GO:1901576 | organic substance biosynthetic process | BP | 2.09E-15 |
| GO:0044238 | primary metabolic process | BP | 6.12E-15 |
| GO:0044237 | cellular metabolic process | BP | 2.79E-14 |
| GO:0009058 | biosynthetic process | BP | 7.46E-14 |
| GO:0071704 | organic substance metabolic process | BP | 8.42E-14 |
| GO:0008152 | metabolic process | BP | 1.35E-07 |
| GO:0009987 | cellular process | BP | 3.31E-05 |
| GO:0006952 | defense response | BP | 3.66E-4 |
| GO:0006216 | cytidine catabolic process | BP | 4.79E-3 |
| GO:0009164 | nucleoside catabolic process | BP | 4.79E-3 |
| GO:0009972 | cytidine deamination | BP | 4.79E-3 |
| GO:0042454 | ribonucleoside catabolic process | BP | 4.79E-3 |
| GO:0046087 | cytidine metabolic process | BP | 4.789E-3 |
| GO:0046133 | pyrimidine ribonucleoside catabolic process | BP | 4.79E-3 |
| GO:0046135 | pyrimidine nucleoside catabolic process | BP | 4.79E-3 |
| GO:0072529 | pyrimidine-containing compound catabolic process | BP | 4.79E-3 |
| GO:1901658 | glycosyl compound catabolic process | BP | 4.79E-3 |
| GO:0000723 | telomere maintenance | BP | 8.04E-3 |
| GO:0032200 | telomere organization | BP | 8.04E-3 |
| GO:0060249 | anatomical structure homeostasis | BP | 8.04E-3 |
| GO:0003333 | amino acid transmembrane transport | BP | 1.53E-2 |
| GO:0006865 | amino acid transport | BP | 1.53E-2 |
| GO:0098656 | anion transmembrane transport | BP | 1.53E-2 |
| GO:1903825 | organic acid transmembrane transport | BP | 1.53E-2 |
| GO:0009082 | branched-chain amino acid biosynthetic process | BP | 3.58E-2 |
| GO:0051259 | protein oligomerization | BP | 4.16E-2 |
| GO:0051260 | protein homooligomerization | BP | 4.16E-2 |
| GO:0003964 | RNA-directed DNA polymerase activity | MF | 3.14E-92 |
| GO:0034061 | DNA polymerase activity | MF | 1.16E-87 |
| GO:0016779 | nucleotidyltransferase activity | MF | 1.29E-68 |
| GO:0004523 | RNA-DNA hybrid ribonuclease activity | MF | 5.01E-65 |
| GO:0016891 | endoribonuclease activity, producing 5'-phosphomonoesters | MF | 3.25E-57 |
| GO:0004521 | endoribonuclease activity | MF | 6.63E-56 |
| GO:0016893 | endonuclease activity, active with either ribo- or deoxyribonucleic acids and producing 5'-phosphomonoesters | MF | 2.21E-55 |
| GO:0004540 | ribonuclease activity | MF | 2.71E-51 |
| GO:0004519 | endonuclease activity | MF | 6.79E-48 |
| GO:0003723 | RNA binding | MF | 4.63E-43 |
| GO:0004518 | nuclease activity | MF | 3.57E-34 |
| GO:0003676 | nucleic acid binding | MF | 4.34E-33 |
| GO:0016772 | transferase activity, transferring phosphorus-containing groups | MF | 1.33E-22 |
| GO:0016788 | hydrolase activity, acting on ester bonds | MF | 6.54E-15 |
| GO:1901363 | heterocyclic compound binding | MF | 1.21E-11 |
| GO:0097159 | organic cyclic compound binding | MF | 1.26E-11 |
| GO:0016740 | transferase activity | MF | 3.51E-11 |
| GO:0005488 | binding | MF | 3.79E-11 |
| GO:0043531 | ADP binding | MF | 5.61E-05 |
| GO:0004455 | ketol-acid reductoisomerase activity | MF | 1.08E-3 |
| GO:0004126 | cytidine deaminase activity | MF | 4.32E-3 |
| GO:0008440 | inositol-1,4,5-trisphosphate 3-kinase activity | MF | 4.32E-3 |
| GO:0008234 | cysteine-type peptidase activity | MF | 7.21E-3 |
| GO:0015171 | amino acid transmembrane transporter activity | MF | 8.21E-3 |
| GO:0008514 | organic anion transmembrane transporter activity | MF | 8.57E-3 |
| GO:0005507 | copper ion binding | MF | 1.15E-2 |
| GO:0046983 | protein dimerization activity | MF | 2.12E-2 |
| GO:1901505 | carbohydrate derivative transporter activity | MF | 2.39E-2 |
| GO:0005342 | organic acid transmembrane transporter activity | MF | 2.52E-2 |
| GO:0046943 | carboxylic acid transmembrane transporter activity | MF | 2.52E-2 |
| GO:0009055 | electron carrier activity | MF | 3.75E-2 |
| GO:0019239 | deaminase activity | MF | 3.78E-2 |
| GO:0008601 | protein phosphatase type 2A regulator activity | MF | 4.94E-2 |
| GO:0000159 | protein phosphatase type 2A complex | CC | 6.99E-3 |
| GO:0008287 | protein serine/threonine phosphatase complex | CC | 6.99E-3 |
| GO:1903293 | phosphatase complex | CC | 6.99E-3 |
| GO:0005811 | lipid particle | CC | 9.34E-3 |
| GO:0012511 | monolayer-surrounded lipid storage body | CC | 9.34E-3 |
| GO:0005839 | proteasome core complex | CC | 3.85E-2 |
| GO:0005634 | nucleus | CC | 4.73E-2 |

*Table S14 Enriched KEGG pathways in expanded gene families in* Apostasia

| Map ID | Map Title | *P* value |
| --- | --- | --- |
| map03018 | RNA degradation | 1.30E-5 |
| map03008 | Ribosome biogenesis in eukaryotes | 1.53E-5 |
| map03015 | mRNA surveillance pathway | 5.84E-5 |
| map00010 | Glycolysis / Gluconeogenesis | 9.02E-5 |
| map03013 | RNA transport | 2.77E-4 |
| map00052 | Galactose metabolism | 2.54E-3 |
| map04626 | Plant-pathogen interaction | 3.67E-3 |
| map03440 | Homologous recombination | 6.44E-3 |
| map00240 | Pyrimidine metabolism | 1.27E-2 |
| map00250 | Alanine, aspartate and glutamate metabolism | 1.42E-2 |
| map00350 | Tyrosine metabolism | 2.19E-2 |
| map00402 | Benzoxazinoid biosynthesis | 4E-2 |

Table S15 Enriched GO terms in significantly expanded gene families in *A. ramifera*

| GO ID | GO term | GO class | *P*-value |
| --- | --- | --- | --- |
| GO:0050660 | flavin adenine dinucleotide binding | MF | 5.95E-17 |
| GO:0008762 | UDP-N-acetylmuramate dehydrogenase activity | MF | 2.10E-16 |
| GO:0050662 | coenzyme binding | MF | 8.68E-15 |
| GO:0043168 | anion binding | MF | 1.61E-07 |
| GO:0000166 | nucleotide binding | MF | 6.78E-07 |
| GO:0055114 | oxidation-reduction process | BP | 9.74E-07 |
| GO:0016491 | oxidoreductase activity | MF | 9.86E-07 |
| GO:0009616 | virus induced gene silencing | BP | 3.90E-05 |
| GO:0043167 | ion binding | MF | 5.64E-05 |
| GO:0004713 | protein tyrosine kinase activity | MF | 0.000187 |

Table S16 Enriched KEGG pathways in significantly expanded gene families in *A. ramifera*

| MapID | MapTitle | *P-*value |
| --- | --- | --- |
| map00908 | Zeatin biosynthesis | 4.31E-07 |
| map00564 | Glycerophospholipid metabolism | 3.55E-06 |
| map00565 | Ether lipid metabolism | 7.79E-06 |
| map04144 | Endocytosis | 0.001193 |

Table S17 Enriched GO terms in significantly contracted gene families in *A. ramifera*

| GO ID | GO term | GO class | *P*-value |
| --- | --- | --- | --- |
| GO:0016772 | transferase activity, transferring phosphorus-containing groups | MF | 4.80E-40 |
| GO:0016740 | transferase activity | MF | 6.46E-31 |
| GO:0004713 | protein tyrosine kinase activity | MF | 2.64E-27 |
| GO:0006468 | protein phosphorylation | BP | 2.49E-25 |
| GO:0004672 | protein kinase activity | MF | 3.17E-25 |
| GO:0044238 | primary metabolic process | BP | 1.59E-21 |
| GO:0044260 | cellular macromolecule metabolic process | BP | 6.35E-21 |
| GO:0071704 | organic substance metabolic process | BP | 1.02E-20 |
| GO:1901363 | heterocyclic compound binding | MF | 3.82E-20 |
| GO:0097159 | organic cyclic compound binding | MF | 3.88E-20 |
| GO:0003824 | catalytic activity | MF | 4.18E-20 |
| GO:0007154 | cell communication | BP | 1.61E-19 |
| GO:0003964 | RNA-directed DNA polymerase activity | MF | 5.51E-19 |
| GO:0006278 | RNA-dependent DNA replication | BP | 5.51E-19 |
| GO:0005524 | ATP binding | MF | 6.00E-18 |
| GO:0044237 | cellular metabolic process | BP | 7.29E-18 |
| GO:0008152 | metabolic process | BP | 2.07E-17 |
| GO:0016779 | nucleotidyltransferase activity | MF | 3.45E-17 |
| GO:0000166 | nucleotide binding | MF | 6.91E-16 |
| GO:0005488 | binding | MF | 1.49E-14 |
| GO:0048544 | recognition of pollen | BP | 1.12E-13 |
| GO:0003723 | RNA binding | MF | 5.75E-13 |
| GO:0006259 | DNA metabolic process | BP | 3.71E-12 |
| GO:0007165 | signal transduction | BP | 2.61E-10 |
| GO:0043167 | ion binding | MF | 7.91E-09 |
| GO:0030246 | carbohydrate binding | MF | 4.20E-08 |
| GO:0015074 | DNA integration | BP | 1.22E-07 |
| GO:0003676 | nucleic acid binding | MF | 5.88E-06 |
| GO:0004553 | hydrolase activity, hydrolyzing O-glycosyl compounds | MF | 7.44E-06 |
| GO:0090304 | nucleic acid metabolic process | BP | 1.19E-05 |
| GO:0034645 | cellular macromolecule biosynthetic process | BP | 2.12E-05 |
| GO:0006807 | nitrogen compound metabolic process | BP | 8.15E-05 |
| GO:0044763 | single-organism cellular process | BP | 0.000109 |

Table S18 Enriched KEGG pathways in significantly contracted gene families in *A. ramifera*

| MapID | MapTitle | *P*-value |
| --- | --- | --- |
| map03440 | Homologous recombination | 1.11E-21 |
| map00604 | Glycosphingolipid biosynthesis - ganglio series | 2.84E-16 |
| map00531 | Glycosaminoglycan degradation | 1.49E-13 |
| map00600 | Sphingolipid metabolism | 3.73E-12 |
| map00511 | Other glycan degradation | 3.86E-11 |
| map00052 | Galactose metabolism | 5.15E-11 |
| map00943 | Isoflavonoid biosynthesis | 0.000583 |
| map04626 | Plant-pathogen interaction | 0.002006 |

Table S19 Number of genes in *MADS*-box gene subfamilies in orchids

|  | *A. ramifera* | *A. shenzhenica* [1] | *P. equestris* [1] | *D. catenatum* [2] | *V. planifolia* |
| --- | --- | --- | --- | --- | --- |
| Type II *MADS*-box genes | | | | | |
| Total | 23 | 27 | 29 | 35 | 30 |
| A | 2 | 2 | 3 | 4 | 1 |
| B | 4 | 4 | 6 | 7 | 4 |
| C/D | 3 | 4 | 5 | 4 | 7 |
| E | 3 | 3 | 6 | 5 | 2 |
| FLC | 0 | 0 | 0 | 0 | 0 |
| MIKC* | 1 | 2 | 1 | 3 | 4 |
| SVP | 3 | 2 | 1 | NA | 4 |
| ANR1 | 4 | 4 | 2 | 3 | 3 |
| SOC1 | 0 | 2 | 2 | 2 | 2 |
| AGL12 | 1 | 1 | 0 | 0 | 2 |
| AGL6 | 1 | 2 | 3 | 3 | 1 |
| Type I *MADS*-box genes | | | | | |
| Total | 7 | 9 | 22 | 28 | 6 |
| Mα | 5 | 5 | 10 | 15 | 4 |
| Mβ | 0 | 0 | 0 | 0 | 0 |
| Mγ | 2 | 4 | 12 | 13 | 2 |

Table S20 Number of genes in *TPS* gene subfamilies in orchids

| Species | Total | TPS-a | TPS-b | TPS-c | TPS-e | TPS-f | TPS-g |
| --- | --- | --- | --- | --- | --- | --- | --- |
| *A. ramifera* | 6 | 1 | 3 | 0 | 1 | 0 | 1 |
| *A. shenzhenica* | 8 | 3 | 4 | 0 | 1 | 0 | 0 |
| *P. equestris* | 22 | 5 | 7 | 3 | 3 | 4 | 0 |
| *D. catenatum* | 26 | 11 | 12 | 0 | 1 | 2 | 0 |
| *P. aphrodite* | 24 | 5 | 9 | 4 | 3 | 3 | 0 |
| *V. planifolia* | 23 | 7 | 7 | 4 | 1 | 0 | 4 |

Table S21 Number of *LOX1*/*LOX5* homologs in orchids

|  | *A. ramifera* | *D. catenatum* | *P. equestris* | *P. aphrodite* | *V. planifolia* |
| --- | --- | --- | --- | --- | --- |
| *LOX1*/*LOX5* | 1 | 4 | 4 | 3 | 1 |


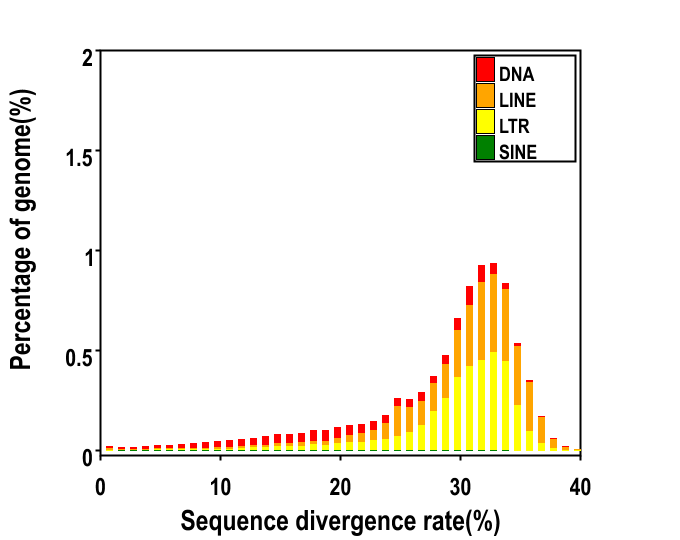


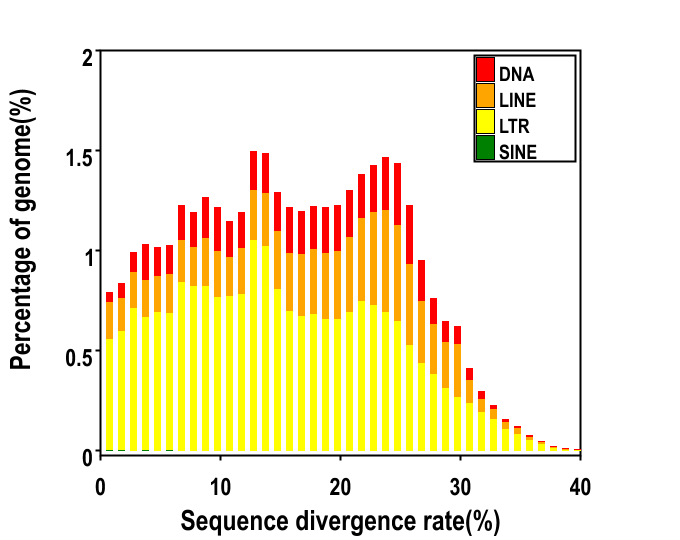


Figure S1 Divergence distribution of four types of transposable elements (TEs) in *A. ramifera*. Upper panel shows TEs identified by RepeatMasker, and bottom panel shows TEs *de novo* identified by LTR-FINDER and RepeatModeler.


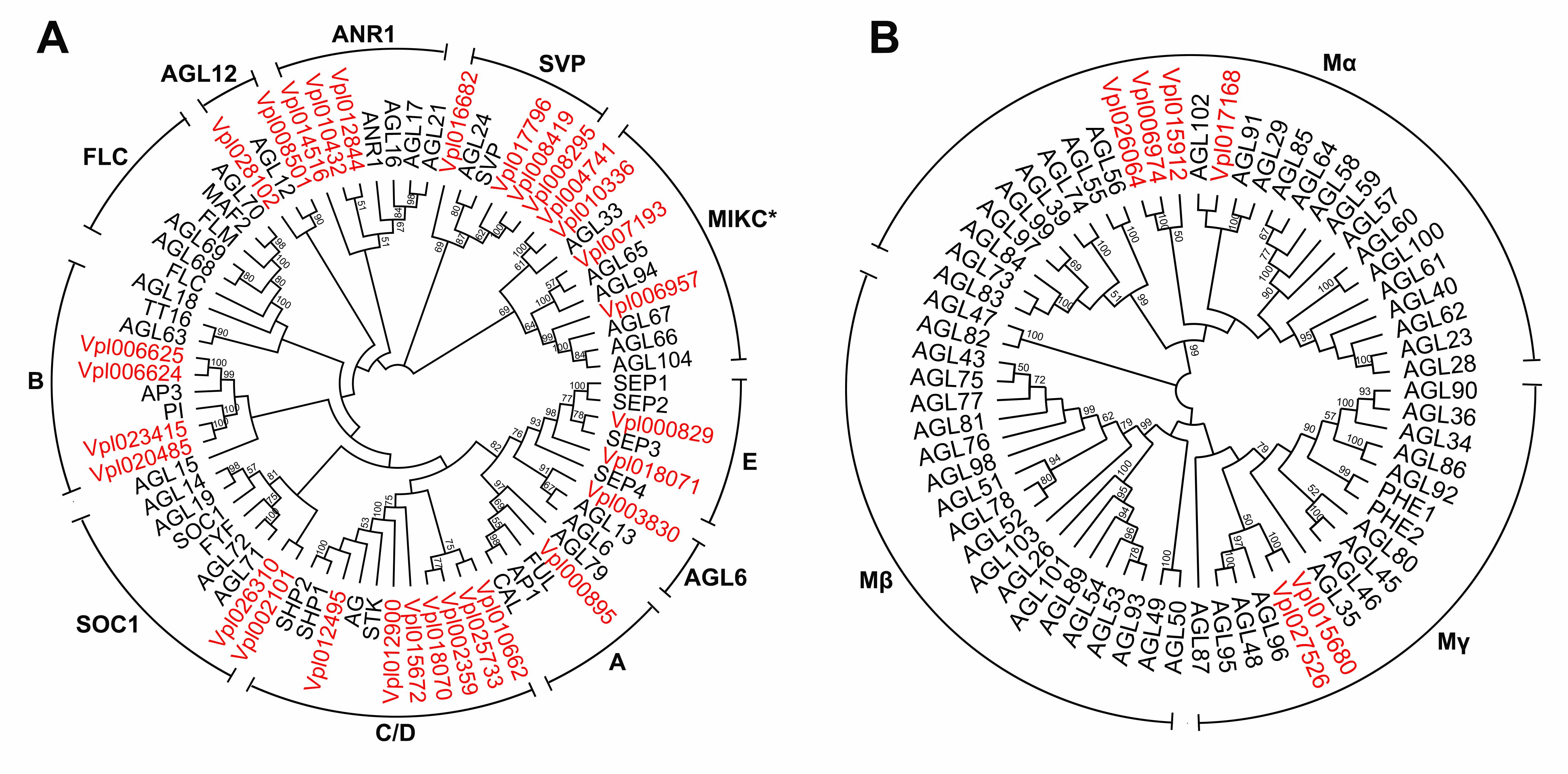


Figure S2 Phylogenetic analysis of *MADS*-box genes in *V. planifolia*. (A) Type II *MADS*-box genes. (B) Type I *MADS*-box genes. Neighbor-joining gene trees were constructed using *MADS*-box genes from *V. planifolia* and *Arabidopsis*. Genes from *V. planifolia* are marked in red. Different *MADS*-box classes are indicated. Numbers above branches are bootstrap support values of at least 50.

**Supplementary References**

1. Zhang GQ, Liu KW, Li Z, Lohaus R, Hsiao YY, Niu SC, et al. The Apostasia genome and the evolution of orchids. Nature. 2017;549(7672):379-83.

2. Zhang GQ, Xu Q, Bian C, Tsai WC, Yeh CM, Liu KW, et al. The Dendrobium catenatum Lindl. genome sequence provides insights into polysaccharide synthase, floral development and adaptive evolution. Sci Rep. 2016;6:19029.
